# Exendin-4 ameliorates cardiac ischemia/reperfusion injury via caveolae and caveolins-3

**DOI:** 10.1186/s12933-014-0132-9

**Published:** 2014-09-07

**Authors:** Yasuo M Tsutsumi, Rie Tsutsumi, Eisuke Hamaguchi, Yoko Sakai, Asuka Kasai, Yoshihiro Ishikawa, Utako Yokoyama, Katsuya Tanaka

**Affiliations:** Department of Anesthesiology, University of Tokushima, 3-18-15 Kuramoto, Tokushima Japan; Department of Nutrition, University of Tokushima, Tokushima, Japan; Cardiovascular Research Institute, Yokohama City University, Yokohama, Japan

**Keywords:** Cardiac protection, Subcellular microdomain, Glucagon-like peptide-1 receptor, Incretin

## Abstract

**Background:**

Exendin-4, an exogenous glucagon-like peptide-1 receptor (GLP-1R) agonist, protects the heart from ischemia/reperfusion injury. However, the mechanisms for this protection are poorly understood. Caveolae, sarcolemmal invaginations, and caveolins, scaffolding proteins in caveolae, localize molecules involved in cardiac protection. We tested the hypothesis that caveolae and caveolins are essential for exendin-4 induced cardiac protection using *in vitro* and *in vivo* studies in control and caveolin-3 (Cav-3) knockout mice (Cav-3 KO).

**Methods:**

Myocytes were treated with exendin-4 and then incubated with methyl-β-cyclodextrin (MβCD) to disrupt caveolae formation. This was then followed by simulated ischemia/reperfusion (SI/R). In addition, cardiac protection *in vivo* was assessed by measuring infarct size and cardiac troponin levels.

**Results:**

Exendin-4 protected cardiac myocytes (CM) from SI/R [35.6 ± 12.6% vs. 64.4 ± 18.0% cell death, P = 0.034] and apoptosis but this protection was abolished by MβCD (71.8 ± 10.8% cell death, P = 0.004). Furthermore, Cav-3/GLP-1R co-localization was observed and membrane fractionation by sucrose density gradient centrifugation of CM treated with MβCD + exendin-4 revealed that buoyant (caveolae enriched) fractions decreased Cav-3 compared to CM treated with exendin-4 exclusively. Furthermore*,* exendin-4 induced a reduction in infarct size and cardiac troponin relative to control (infarct size: 25.1 ± 8.2% vs. 41.4 ± 4.1%, P < 0.001; troponin: 36.9 ± 14.2 vs. 101.1 ± 22.3 ng/ml, P < 0.001). However, exendin-4 induced cardiac protection was abolished in Cav-3 KO mice (infarct size: 43.0 ± 6.4%, P < 0.001; troponin: 96.8 ± 26.6 ng/ml, P = 0.001).

**Conclusions:**

We conclude that caveolae and caveolin-3 are critical for exendin-4 induced protection of the heart from ischemia/reperfusion injury.

## Introduction

Glucagon-like peptide-1 (GLP-1) is an intestinal hormone secreted in a nutrient-dependent manner that stimulates insulin secretion and inhibits glucagon secretion and gastric emptying, resulting in reduced post-prandial hyperglycemia [1]. GLP-1 acts upon the GLP-1 receptor (GLP-1R), which belongs to the family of G-protein-coupled receptor (GPCRs) [2]. This receptor is abundantly expressed in the gastrointestinal tract, but has also been detected in the central nervous system, heart, vascular smooth muscle cells, endothelial cells, and macrophages [3,4]. Recently, GLP-1 has been shown to reduce an infarct size in both *in vitro* and *in vivo* animal models of cardiac ischemia/reperfusion injury [5–7] and exendin-4 (Ex-4), an exogenous GLP-1R agonist isolated form the Gila monster lizard [8], has reported to have very similar effects [4,9,10].

Caveolae are small flask-like invaginations of sarcolemmal membrane that are enriched in lipids. Caveolin-3 (Cav-3) is the principal protein component of caveolae and can interact with a number of signaling molecules including G protein, receptor tyrosine kinases, and GPCRs *via* caveolin-binding motif [11–13]. In our previous studies, we have shown that both caveolae and Cav-3 were essential in cardiac protection against ischemia/reperfusion in the animal model [14–17]. However, studies addressing the plasma-membrane localization of GLP-1R are not fully known and the impact of caveolae and Cav-3 on GLP-1-induced cardiac protection has not been investigated. Therefore, we hypothesized that both caveolae and Cav-3 are a critical component of GLP-1-induced cardiac protection and that coordination of protective signaling is dependent on the co-localization of Cav-3 and GLP-1R.

## Material and methods

All animals were treated in compliance with the Guidelines for Proper Conduct of Animal Experiment and Related Activities (Ministry of Education, Culture, Sports, Science and Technology of Japan) and the protocols, which was assigned to ARRIVE guidelines [18], approved by the Animal Care and Use Committee at the University of Tokushima. Male Wistar rats (12–14 weeks old, 250–300 g body weight) and male C57BL/6 mice (8–10 weeks old, 21–25 g body weight) were purchased from Japan SLC, and Cav-3 KO mice (8–10 weeks old, 21–25 g body weight) were created as reported previously [19]. The animals were kept on a 12 hour light–dark cycle in a temperature and humidity-controlled room, and had *ad lib* access to food and water.

### Preparation of Cardiac Myocytes (CM)

CM were isolated from adult male Wistar rats as described [20,21]. In brief, hearts were retrograde perfused on a Langendorff apparatus and digested with collagenase (Worthington). Myocytes were plated in Medium 199 (4% fetal bovine serum and 1% penicillin/streptomycin) on laminin (2 μg/cm^2^)-coated plates for 1 h. Plating media was changed to serum-free media (1% bovine serum albumin) to remove non-myocytes and CM were incubated for 24 h at 37°C in 5% CO_2_.

### Simulated ischemia/reperfusion (SI/R) in isolated cardiac myocytes

CM were plated on laminin-coated 12-well plates, and simulated ischemia was induced by replacing the air content with a 95% N_2_ and 5% CO_2_ gas mixture at 2 L/min in a chamber and by replacing the media to glucose-free media for 60 min. This was then followed by 60 min of “reperfusion” by replacing the media with normal maintenance media and by incubating the cells with 21% O_2_ and 5% CO_2_ [16]. CM were exposed to 0.3 nM or 3.0 nM Ex-4, a GLP-1R agonist, for 1 h prior to SI/R. Cell death was quantified by counting trypan blue-stained cells with results expressed as a percentage of total cells counted. Cells were counted (3 random fields per well) using ImageJ software to determine percent cell death. To determine the impact of caveolae on cardiac protection, methyl-β-cyclodextrin (MβCD) was used as described [16]. CM were incubated under maintenance media (control conditions) or in the presence of MβCD (1 mM) for 1 h before SI/R.

### Depolarization of the mitochondrial membrane

To analyze mitochondrial membrane potential, we used the JC-1 dye (MitoPT JC-1, ImmunoChemistry Technologies, Bloomington, MN), which shifts the fluorescence emission from red (580 nm) to green (488 nm) as mitochondrial membrane is depolarized. After SI/R, as described above, myocytes were incubated with JC-1 for 20 min at 37°C, and cellular fluorescence was determined by a fluorescence microscope (Leica TCS NT, Heidelberg, Germany). Data are assessed by comparing the ratios of red/green.

### Gene expression analyses

Total RNA was extracted from CM using RNeasy Plus Universal Mini Kits (QIAGEN, Valencia, CA). Total RNA (1 μg) was reverse-transcribed to cDNA in a final volume of 20 μL using the Primescript RT Reagent kit (Takara, Shiga, Japan). Real-time polymerase chain reaction (PCR) was performed in a final volume of 10 μL containing 50 ng of the cDNA template and primers using a StepOnePlus Real-Time PCR System (Life Technologies, Carlsbad, CA). To determine the effect on apoptosis gene expression, we measured the expression of the BH3-interacting domain death agonist (BID), Bcl-2-associated death promoter (BAD), Caspase-3, Caspase-8, and Caspase-9, and Bcl-2 associated X protein (BAX) genes. To determine the effect on anti-apoptosis gene expression, we measured the expression of the B-cell lymphoma 2 (BCL-2) and inhibitor of apoptosis 1 (IAP-1) genes.

### Immunofluorescence

CM were fixed with paraformaldehyde, incubated with 100 mM glycine, permeabilized in 0.1% buffered Triton X-100, and blocked with 1% bovine serum albumin, phosphate-buffered saline, and 0.05% Tween. Samples were then incubated with primary antibody (GLP-1R and caveolins-3, Santa Cruz Biotechnology, Santa Cruz, CA) (1:100) in 1% bovine serum albumin, phosphate-buffered saline, and 0.05% Tween for 24 h. Excess antibody was removed, and samples were incubated with fluorescein Alexa-conjugated secondary antibodies (1:250) for 1 h. To remove excess secondary antibody, samples were washed with phosphate-buffered saline/0.1% Tween and samples were mounted in UltraCruz (Santa Cruz Bioctechnology) for microscopy imaging. Fluorescent images of cell sections excited at 488 and 560 nm were captured using a confocal laser scanning microscope (Leica TCS NT, Heidelberg, Germany) equipped with an argon-krypton laser source. Images were taken at 400 × magnification and were assessed quantitatively by Image-Pro Plus (Media Cybernetics, Silver Spring, MD).

### Sucrose density fractionation

Whole left ventricles or myocytes were used for sucrose density membrane fractions as reported previously [22]. Briefly, approximately 1 ml of lysate was mixed with 1 ml of 80% sucrose in 25 mM MES and 150 mM NaCl (MES buffered saline, MBS, pH 6.5) to form 40% sucrose and loaded at the bottom of an ultracentrifuge tube. A discontinuous sucrose gradient was generated by layering 6 ml of 35% sucrose prepared in MBS followed by 4 ml of 5% sucrose in MBS. The gradient was centrifuged at 175,000 g using a P70AT2 rotor (Hitachi Koki Co.) for 3 h at 4°C. After centrifugation, samples were removed in 1 ml aliquots to yield 12 fractions. We defined fraction 4–6 as buoyant membrane fractions enriched in caveolae and proteins associated with caveolae. Fraction 9–12 were defined as nonbuoyant fractions.

### Immunoprecipitation

Immunoprecipitation was performed using Protein A Sepharose CL-4B (GE Healthcare) as described previously [23]. Buoyant fraction samples were incubated with primary antibody (GLP-1R and caveolins-3, Santa Cruz Biotechnology) for 3 h at 4°C, immune-precipitated overnight with protein-agarose at 4°C, and then centrifuged for 5 min at 13,000 g. Protein-agarose pellets were washed 3 times. Wash buffer was removed and sample buffer was added, and then boiled for 5 min at 95°C for immunoblotting.

### Immunoblot analysis

Proteins were separated by SDS-PAGE 10% polyacrylamide precast gels (Bio-Rad Laboratories) and transferred to a polyvinylidene diflouride membrane by electroelution. Membranes were blocked in PBS containing 2.0% nonfat dry milk and incubated with primary antibody overnight (GLP-1R and caveolins-3, Santa Cruz Biotechnology; GAPDH, Santa Cruz Biotechnology and Cell Signaling Technology) and at 4°C. Bound primary antibodies were visualized using secondary antibodies (Santa Cruz Biotechnology) conjugated with horseradish peroxidase from Santa Cruz Biotechnology and ECL reagent from GE Healthcare [24]. All displayed bands migrated at the appropriate size, as determined by comparison to molecular weight standards (Santa Cruz Biotechnology).

### Ischemia/reperfusion protocol and experimental groups

C57BL/6 mice and Cav-3 knockout (Cav-3 KO) micewere anesthetized with pentobarbital sodium (80 mg/kg ip) and mechanically ventilated by using a pressure-controlled ventilator (TOPO Ventilator, Kent Scientific) as described before [25]. Core temperature was maintained with a heating pad and ECG leads were placed to record heart rate. The hemodynamic effects were measured through the right carotid artery cannulation with a 1.4 F Mikro-tip pressure transducer (Model SPR-671, Millar Instruments), which was connected to an amplifier (Model TC-510, Millar Instruments) for determination of heart rate, arterial blood pressure, and rate pressure product as previous before [26]. After thoracotomy, baseline was established, and mice were randomly assigned to experimental protocols. Lethal ischemia was produced by occluding the left coronary artery with a 7–0 silk suture on a taper BV-1 needle (Ethicon) for 30 min. After 30 min of occlusion, the ligature was released and the heart was reperfused for 2 h. After reperfusion, mice were heparinized, and the coronary artery was again occluded. The area at risk (AAR) was determined by staining with 1% Evans blue (Sigma). The heart was immediately excised and placed into 1% agarose and allowed to harden. Once hardened, the heart was cut into 1.0-mm slices (McIlwain tissue chopper; Brinkmann Instruments). Each slice of left ventricle (LV) was then counterstained with 2,3,5,-triphenyltetrazolium chloride (Sigma). After overnight storage in 10% formaldehyde, slices were weighed and visualized under a microscope (SZ61-TR, Olympus) equipped with a charge coupled device camera (DXM 1200 F, Nikon). The images were analyzed (Image-Pro Plus, Media Cybernetics), and AAR and infarct size (IS) was determined by planimetry as previously described [27,28]. Cardiac troponin I levels in the serum were measured using a High Sensitivity Mouse Cardiac Troponin-I ELISA Kit (Life Diagnostics).

### Statistical analysis

Statistical analyses were performed by one-way and two-way ANOVA for repeated measures, followed by Bonferroni post-hoc test. All data are expressed as mean ± SD. Statistical significance was defined as *P* < 0.05.

## Results

### Experimental animals

The animals’ health status was monitored throughout the experiments by a health surveillance program. A total of 98 animals were used in the experiments described here (35 animals for *in vitro* simulated ischemia/reperfusion, 23 for immunofluorescence and immunoblot analyses, and 40 for in vivo ischemia/reperfusion experiments). Five mice died shortly after ischemia/reperfusion because of fatal cardiac arrhythmia in the *in vivo* experiments (control, 1; Ex-4 administration, 2; Cav-3 KO control, 1; Cav-3 KO Ex-4 administration, 1).

### Exendin-4 induces cardiac protection in CM

**Figure 1:**
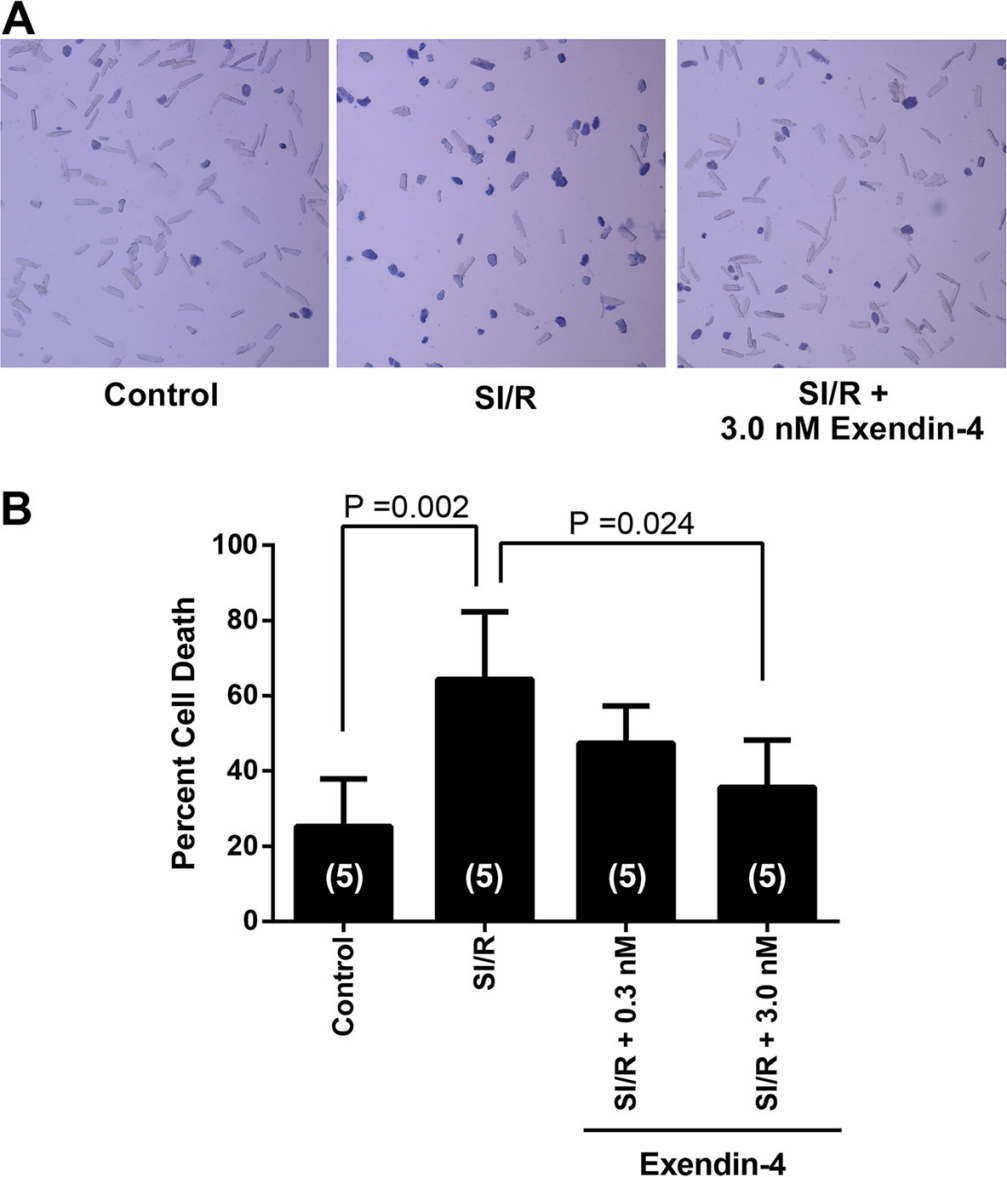
**Effects of exendin-4 on simulated ischemia/reperfusion (SI/R) of cardiac myocytes. (A)** Cardiac myocytes were rod-shaped and treated with various concentration of exendin-4 prior to exposure to SI/R. **(B)** Cell death was determined by trypan blue staining. Optimal protection was observed at 3.0 nM exendin-4. Group sizes are indicated on the individual bars in parentheses.

CM were administered with various concentration of Ex-4 and then SI/R (Figure 1A). Administration of 0.3 nM and 3.0 nM Ex-4 before SI/R decreased cell death when compared to SI/R alone (47.4 ± 9.9%, and 35.6 ± 12.6% and 64.4 ± 18.0% cell death, respectively, n = 5 per each groups; Figure 1B).

### MβCD abolish exendin-4 induced cardiac protection

**Figure 2:**
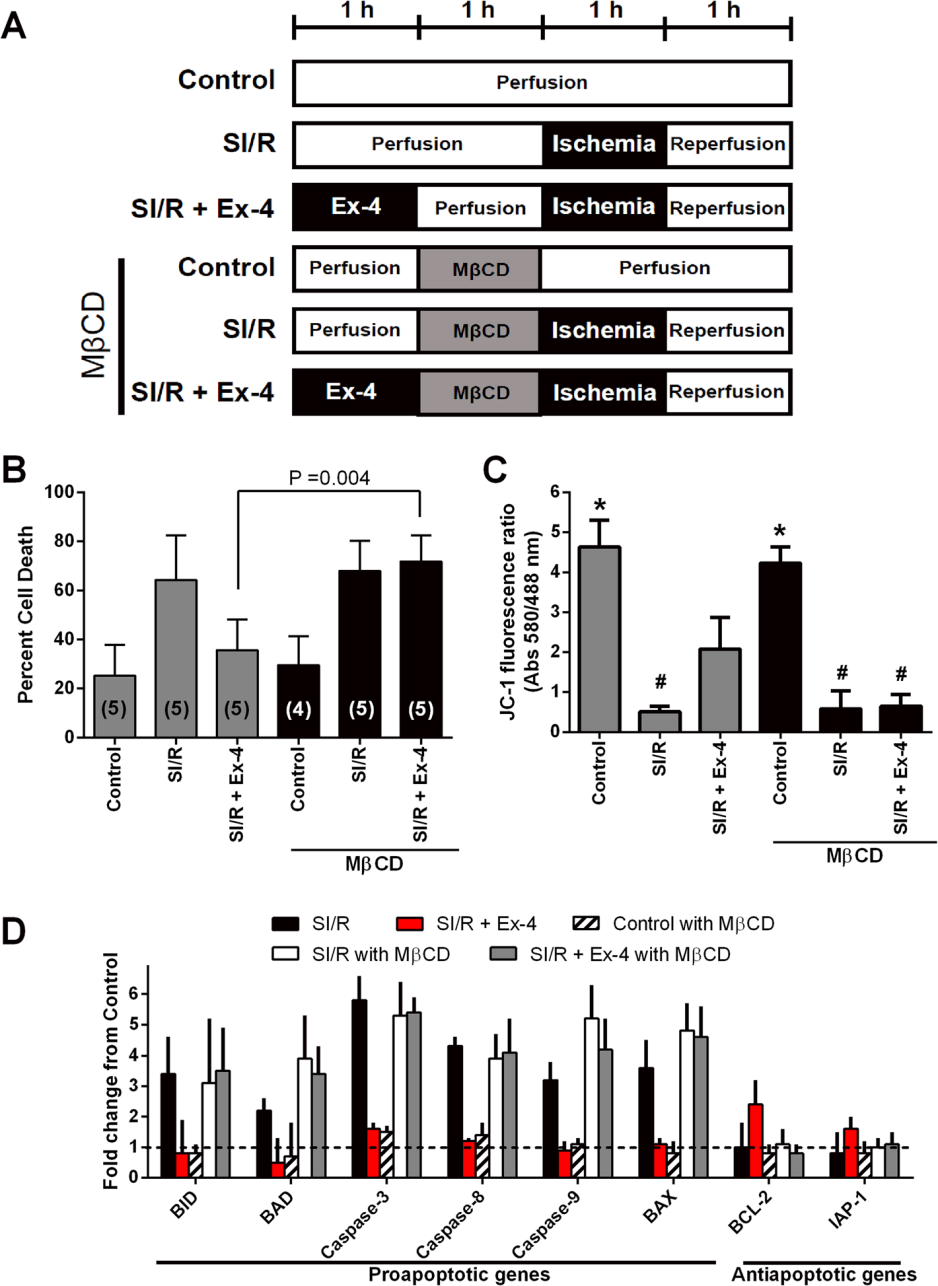
***In vitro*** **assessment of the role of caveolae in exendin-4 (Ex-4) induced cardiac protection. (A)** Summary illustration of *in vitro* experimental groups. **(B)** Cardiac myocytes exposed to simulated ischemia/reperfusion (SI/R) were exposed to experimental procedures outlined in A. Cell death was determined by trypan blue staining. Cardiac myocytes under control conditions (Control) had minimal cell death. Methyl-β-cyclodextrin (MβCD) abolished the Ex-4 induced cardiac protection effect. Group sizes are indicated on the individual bars in parentheses. **(C)** Apoptotic changes were measured by investigating mitochondrial membrane potential using JC-1 after SI/R. The excitation rate (red/green) indicates changes within the mitochondrial membrane potential. *P < 0.001 vs. SI/R, SIR + Ex-4, SI/R with MβCD, and SI/R + Ex-4 with MβCD. #P < 0.05 vs. Control, SI/R + Ex-4, and Control with MβCD. n = 4 per each group. **(D)** Real-time polymerase chain reaction analysis of pro-apoptotic and anti-apoptotic gene expression after re-oxygenation. n = 4 per each group.

CM were incubated with 1% BSA with 0.1% penicillin/streptomycin (Control) or in control media along with 3.0 nM Ex-4, and/or then incubated with 1 mM MβCD (Figure 2A). In the present study, the protective effect of Ex-4 was abolished in CM with MβCD (35.6 ± 12.6% [n = 4] and 71.8 ± 10.8% [n = 5] cell death, respectively; P = 0.004). Additionally, we observed no significant increase in basal cell death with the various treatments (Figure 2B).

To test whether Ex-4 inhibited apoptosis by modifying the mitochondrial membrane potential during reperfusion injury, we measured a membrane potential-sensitive dye, 5,5’,6,6’-tetrachloro-1,1’,3,3’-tetraethylbenzamidazolocarbocyanin iodide (JC-1). As shown in Figure 2C, Ex-4 inhibited reduction of mitochondrial membrane potential that occurred in the re-oxygenated cells expressing SI/R suggesting inhibition of apoptosis. This was further confirmed as Ex-4 decreased pro-apoptic and increased anti-apoptotic gene expression (Figure 2D; n = 4 per each groups).

### Co-localization between GLP-1R and Cav-3, and MβCD alter caveolins expression

**Figure 3:**
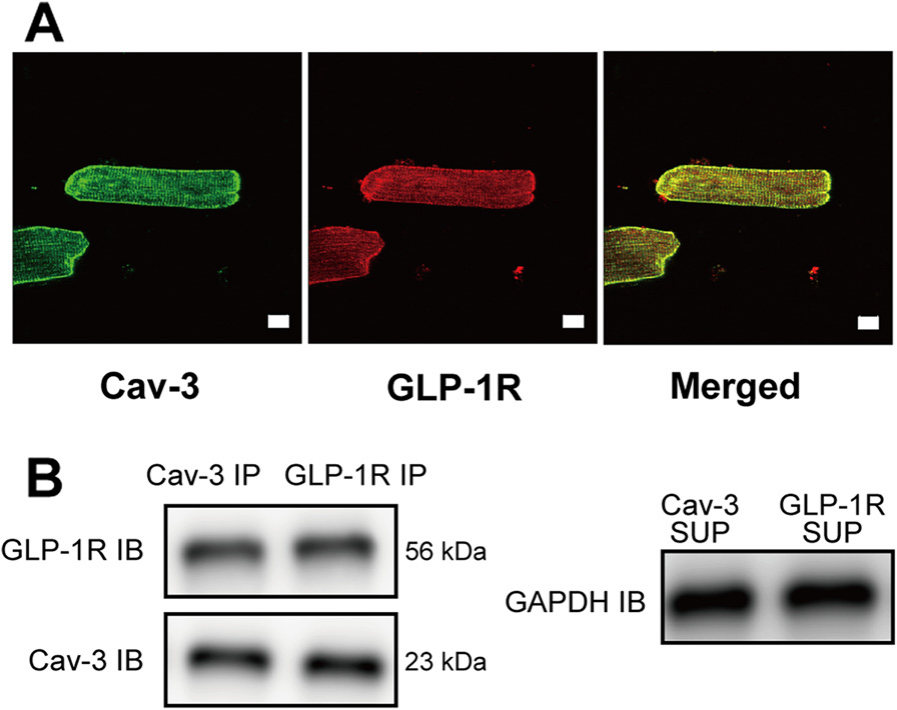
**Glucagon-like peptide-1 receptor (GLP-1R) localization with caveolins or caveolae. (A)** Immunofluorescence analysis of the expression and colocalization of caveolin-3 (Cav-3) and GLP-1R in cardiac myocytes. Fluorescent secondary antibodies were used to determine Cav-3 (green) and GLP-1R (red) localization, and strong colocalization (merged images, yellow) were observed on the cell surface membrane. Bar length = 10 μm. **(B)** The colocalization was confirmed by immunoprecipitation (IP). Immunoblot (IB) analysis detected Cav-3 and GLP-1R in Cav-3 and GLP-1R IP of cell lysates. Supernatants (SUP) from which the immunoprecipitates were generated were stained for GAPDH as loading controls.

**Figure 4:**
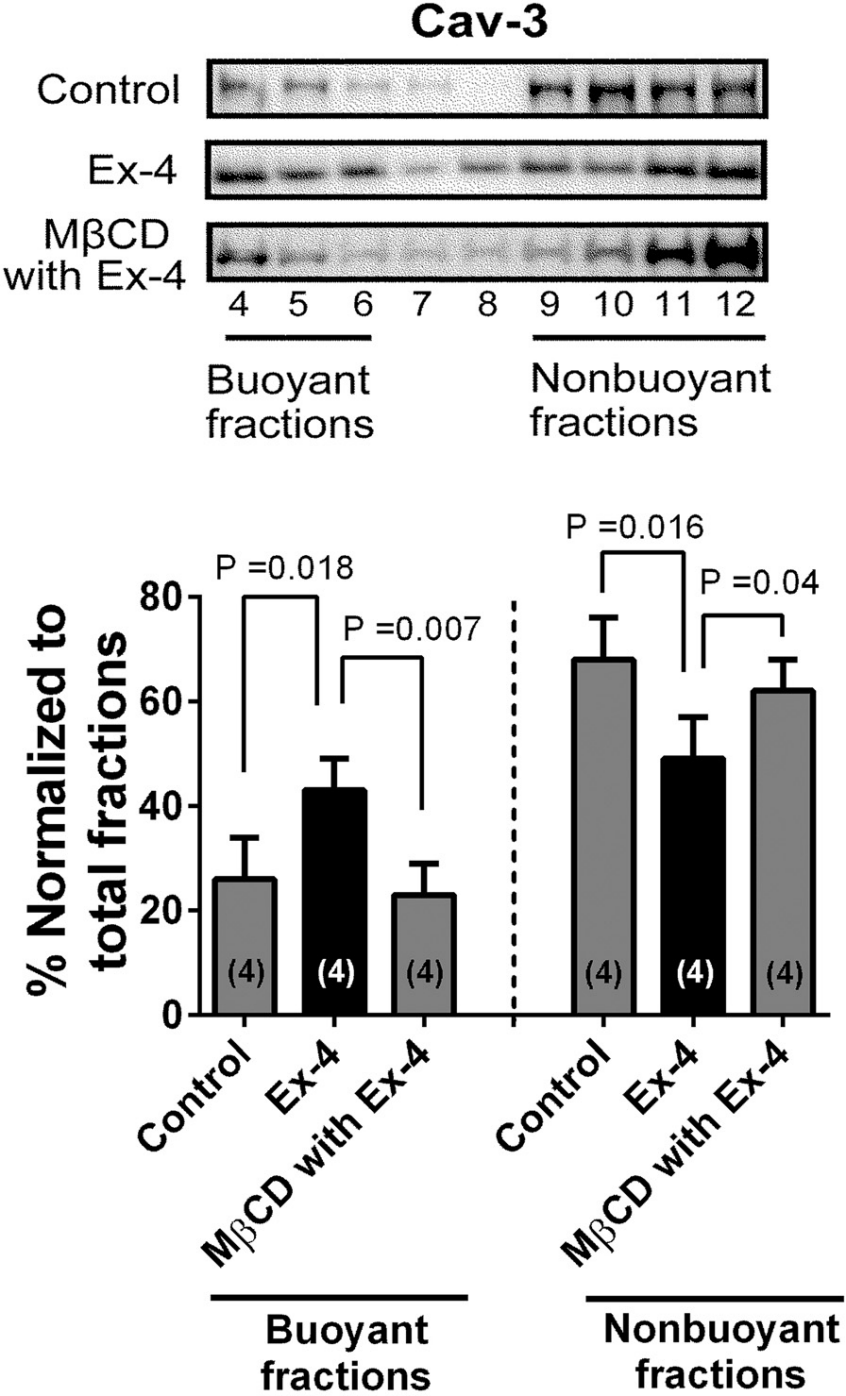
**Lysed and fractionated hearts on sucrose density gradient.** Fractions were collected and probed for caveolin-3 (Cav-3). We defined fractions 4–6 as buoyant membrane fractions enriched in caveolae and proteins associated with caveolae; fractions 9–12 were defined as nonbuoyant fractions, noncaveolar membranes. Fraction 7–8 were considered a transition zone and were not analyzed. Significant localization of Cav-3 in buoyant fractions was observed in the groups treated with exendin-4 (Ex-4), whereas control and methyl-β-cyclodextrin (MβCD)-treated with Ex-4 cells showed no effects on Cav-3 localization. Group sizes are indicated on the individual bars in parentheses.

Immunofluorescence microscopy showed that Cav-3 co-localizes with GLP-1R on the surface of the CM (Figure 3A). Co-immunoprecipitation experiments using cardiac lysates and antibodies to Cav-3 and GLP-1R provided further evidence for the interaction of these proteins (Figure 3B). Expression of Cav-3 in buoyant caveolar fractions (fractions 4–6) was significantly increased after administration of Ex-4 as compared with control mice, and Ex-4 induced migration of Cav-3 from non-buoyant to buoyant fraction was eliminated by MβCD (Figure 4).

### Caveolin-3 is required for exendin-4 induced cardiac protection

**Figure 5:**
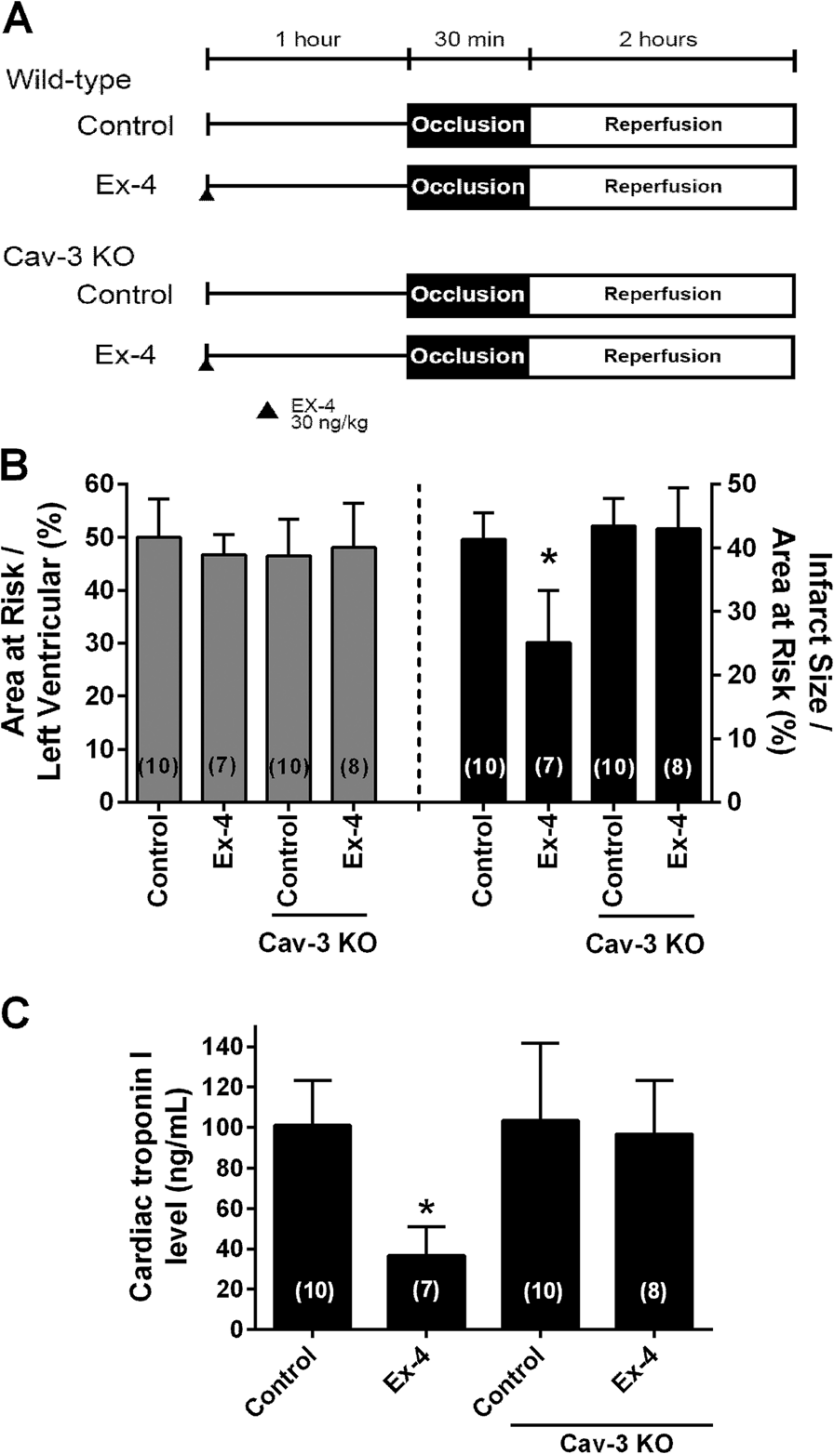
**Caveolin-3 expression and reduction in infarct size.** Mice underwent 30-min coronary artery occlusion followed by 2-h reperfusion after 24-h recovery from pretreatment with oxygen (Control) or exendin-4 (Ex-4) in wild-type and caveolin-3 knockout (Cav-3 KO) mice. **(A)** *In vivo* Ex-4 induced cardiac protection protocol. **(B)** Area at risk was calculated as a percentage of the left ventricle and revealed no significant differences between all groups. Ex-4 induced cardiac protection was abolished Cav-3 KO mice, as shown by no significant decrease in percent infarct size / area at risk when compared to control Cav-3 KO; however, a significant decrease in infarct size was observed between wild-type Ex-4 and Cav-3 KO Ex-4. **(C)** Cardiac troponin I, a marker of myocardial damage also revealed a significant decrease in Ex-4 treated control mice, but no effect in Cav-3 KO mice. Group sizes are indicated on the individual bars in parentheses. *P < 0.001 compared with Cav-3 KO pretreated with Ex-4.

To assess the role of Cav-3 in the protection from ischemia/reperfusion injury, we treated C57BL/6 wild-type mice or Cav-3 KO mice with Ex-4 administration, and then exposed the mice to ischemia/reperfusion (Figure 5A). We found no significant differences between groups in pre-occlusion heart rate, blood pressure, or rate pressure product with and without Ex-4 (Table 1). The ability of Ex-4 to protect from ischemia/reperfusion injury was abolished in Cav-3 KO mice compared to wild-type animals (43.0 ± 6.4% [n = 8] and 25.1 ± 8.2% [n = 7] IS/AAR, P < 0.001) even though there was a similar AAR in all groups of animals (Figure 5B). Cardiac troponin I (cTnI) levels were significantly attenuated by Ex-4 treatment in wild-type mice compared to control mice subjected to ischemia/reperfusion (36.9 ± 14.2 and 101.1 ± 22.3 ng/ml, P < 0.001); however, GLP-1 failed to reduce cTnI in Cav-3 KO mice and a level similar to control Cav-3 KO mice was observed (103.4 ± 38.4 and 96.8 ± 26.6 ng/ml, Figure 5C).

**Table 1:** **Hemodynamics**

	**Baseline**	**Pre-occlusion**	**Ischemia**	**Reperfusion**
			**30 min**	**2 h**
Heart rate, beats · min^−1^				
WT Control	430 ± 26	426 ± 26	383 ± 39*^#^	363 ± 26*^#^
WT Ex-4	432 ± 16	413 ± 41	402 ± 16	398 ± 9
Cav-3 KO Control	413 ± 27	415 ± 25	383 ± 25	381 ± 32
Cav-3 KO Ex-4	416 ± 28	419 ± 26	376 ± 39	357 ± 36*^#^
Mean arterial pressure, mmHg				
WT Control	75 ± 6	75 ± 6	70 ± 6	65 ± 10*^#^
WT Ex-4	78 ± 5	75 ± 5	72 ± 7 70 ± 8	
Cav-3 KO Control	77 ± 4	75 ± 4	71 ± 3* 65 ± 6*^#^	
Cav-3 KO Ex-4	76 ± 5	75 ± 5	70 ± 3	66 ± 5*^#^
Rate-Pressure Product, beats · min^−1^ · mmHg · 10^3^				
WT Control	32.0 ± 3.3	32.0 ± 1.4	26.7 ± 3.5*^#^	23.7 ± 4.7*^#^
WT Ex-4	33.6 ± 2.7	31.2 ± 4.1	29.2 ± 3.5	28.0 ± 3.6*
Cav-3 KO Control	31.9 ± 2.2	31.1 ± 2.6	27.0 ± 2.3*^#^	25.0 ± 3.5*^#^
Cav-3 KO Ex-4	31.5 ± 3.7	31.5 ± 3.2	26.4 ± 3.5*^#^	23.4 ± 2.6*^#^

Data are mean ± SD. Wild-type (WT) or caveolins-3 knockout (Cav-3 KO) mice were randomly exposed to exendin-4 (Ex-4).

^*^Significantly (*P* < 0.05) different from baseline (intragroup comparison).

^#^Significantly (*P* < 0.05) different from pre-occlusion (intragroup comparison).

## Discussion

In the current study, treatment with MβCD, an agent that has been shown to decrease the number of caveolae, produced an attenuation of Ex-4 (GLP-1R agonist) induced cardiac protection in *in vitro* models. Additionally, consistent with these findings, we observed that Ex-4 induced cardiac protection cannot be elicited in Cav-3 KO mice, indicating that the presence of caveolae (dependent on Cav-3 expression) is essential for myocardial protection in the *in vivo* mouse models. This is the first study to investigate the role of caveolins or caveolae in Ex-4 induced cardiac protection.

### GLP-1 cardioprotection

Nutrient-responsive intestinal hormones including GLP-1 are rapidly metabolized by enzyme dipeptidyl-peptidase-4 (DPP-4) to generate an N-terminally truncated metabolite GLP-1 (9–36) [1,29]. Previous studies have demonstrated that the cardioprotective effect of exogenous GLP-1 were attributed to GLP-1R activation and subsequent recruitment of numerous intracellular signaling pathways involving protein kinase B, extracellular regulated kinases, p70S6K, and 5’ adenosine monophosphate-activated protein kinase as well as downstream phosphorylation and inhibition of the pro-apoptotic protein BAD [5,6,30]. Hausenloy *et al*. also showed that chronic treatment with DPP-4 inhibitors reduce infarct size *via* the GLP-1R-protein kinase A pathway, in a glucose dependent manner *in vivo* rat models and confirmed the cardioprotective action of the endogenous intact GLP-1 on ischemia/reperfusion injury [31]. Moreover, Bao *et al*. [32] revealed that the long acting GLP-1R agonist could provide more sustained cardioprotective effect in the setting of acute myocardial ischemia/reperfusion injury than the short-acting Ex-4.

### GLP-1 and the caveolin-dependent pathway

Caveolae, cholesterol and sphingolipid enriched invaginations of plasma membrane play a role physiological functions and vital to cardiac protective mechanisms. Caveolae and caveolins have been shown to play a fundamental role in the phenomenon of myocardial protection against ischemia/reperfusion injury [11–13]. In the present study, we investigated that wild-type mice treated with GLP-1 analogue, Ex-4, were protected against ischemia/reperfusion injury *in vivo*, whereas Cav-3 KO mice were not. In addition, Ex-4 protected isolated CM from hypoxia-induced cell death *in vitro* and had profound effects on membrane microdomains of CM. Our previous studies also revealed that Cav-3 KO mice, which decrease the number of myocardial caveolae, lose the ability to undergo cardioprotection from ischemia/reperfusion injury both *in vitro* and *in vivo* models [14–17]. Although there has been little evidence regarding the relationship between caveolae, caveolins and GLP-1R within the heart, other organ systems including human embryonic kidney (HEK) 293 cells and pancreatic β cells [33,34]. Syme *et al*. demonstrated that GLP-1 receptor interacts with Cav-1 in an association that is necessary for receptor trafficking to the cell membrane and signaling activity in HEK 293 cells [33]. Furthermore, Yang *et al*. demonstrated that activation peroxisome proliferator-activated receptor β/σ protects pancreatic β cells from apoptosis by upregulating the expression of GLP-1R, and sterol regulatory element binding protein-1c/Cav-1 pathway regulates GLP-1R expression [34].

In the present study, we showed that that GLP-1R interacted with Cav-3 and that the administration of Ex-4 led to cardiac protection. Caveolins can interact with a series of signaling molecules, including GPCRs *via* caveolin-binding motifs and may act as a molecular chaperone for GPCRs [12,13]. Overexpression of a dominant-negative form of Cav-1 or mutations within the Cav-1 binding domain of the GLP-1R attenuated GLP-1 binding and GLP-1R expression at the membrane [33]. Collectively, these data implicate that caveolae and caveolins are essential for GLP-1 induced cardiac protection by mediating the GLP-1R.

### Hemodynamic effects of GLP-1

GLP-1 has been shown to increase blood pressure and heart rate in rats [35,36] although others failed to demonstrate any hemodynamic changes in the porcine models [37,38] and human studies [39–42]. In addition, Bose *et al*. investigated that the effects of GLP-1 infusion in rats subjected to 30 min ischemia and 120 min of reperfusion and observed that GLP-1 had no hemodynamic differences in their *in vivo* and *ex vivo* experimental models [5,30,43]. The hemodynamic effects of Ex-4 were also assessed in the animal models, in which dose-dependent increases in mean arterial pressure and heart rate were noted in rats [44]. In our *in vivo* studies, however, there were not any hemodynamic changes among the groups at the pre-occlusion time. This may be due to the dose and the timing of administration, Gardiner *et al*. showed that at a dose of 25 ng/kg i.v., Ex-4 had little effect, but at higher concentrations (250 ng/kg) significant tachycardia and pressor effects were noted for 60 min [44]. As the dose and time period used for cardiac protection in mice are not known, we selected the dosage and time of administration for Ex-4 based on the reports of Gardiner *et al*. to prevent any hemodynamic differences during pre-occlusion (250 ng/kg i.v. at 60 min before occlusion) [44]. Furthermore, GLP-1 has been shown to have central nervous effects on the control of blood pressure and heart rate [45]; however, this mechanism can be avoided in the *in vitro* setting. In our *in vitro* mouse models, we used 3.0 nM Ex-4 concentration, consistent with previous study by Ban *et al*. in which 3.0 nM Ex-4 protect against after ischemia/reperfusion in isolated mouse hearts [4,10].

### Study limitations

There are several limitations in the present study. First, we evaluated the GLP-1R dependent effects of Ex-4 in experiments that investigated ischemia/reperfusion injury. Recent studies suggest that GLP-1 (9–36), the metabolite that is generated by DPP-4 and 1000-fold lower affinity to GLP-1R [46], also improve LV contractile function and post-ischemic myocardial injury [47]. Furthermore, GLP-1R knockout mice have lower heart rate and blood pressure with an increase in cardiac mass and GLP-1 has been shown to protect perfused hearts from rodents lacking GLP-1R from ischemia [4]. These findings suggest that GLP-1 and its metabolite GLP-1 (9–36) may be capable of exerting GLP-1 receptor-independent pathways on the cardiovascular system [10]. Second, Cav-3 KO mice have a variety of deleterious phenotypes (*i.e.*, muscle degeneration, insulin resistance, and progressive cardiomyopathy with age) that may affect outcome after ischemia/reperfusion injury [19,48,49].

### Clinical implications

As a regulator of glucose homeostasis, an exogenous GLP-1 analogue or potentiating endogenous GLP-1 by DPP-4 inhibitors show promise for the treatment of type 2 diabetes mellitus (T2DM) associated with cardiovascular disease. Moreover, there have been several clinical trials using GLP-1 as a therapy for cardiovascular disease in human subjects. Exenatide, an exogenous GLP-1 analogue, was found to be more beneficial than the other current regimens (DPP-4 inhibitors, insulin or tiazolidinediones), in reaching therapeutic goals recommended by the American Diabetes Association in the treatment of T2DM, which is also promising in the reduction of other co-morbidities such as cardiovascular risk [50]. Lonborg *et al*. [51] has shown that exenatide resulted in an increased salvage index among ST-segment elevation myocardial infarction patients with hyperglycemia and normoglycemia. Interestingly, endogenous circulating GLP-1 level was found to be increased in patients with high cardiovascular risk, suggesting it represents a contra-regulatory response in states of increased metabolic risk [52].

## Conclusions

In conclusion, the current results demonstrate that GLP-1R co-localized with caveolae and caveolins-3 are essential for the cardiac protection induced by exendin-4 from ischemia/reperfusion injury.

## Abbreviations

GLP-1R: Glucagon-like peptide-1 receptor
Cav-3: Caveolin-3
Cav-3 KO: Caveolins-3 knockout
MβCD: Methyl-β-cyclodextrin
SI/R: Simulated ischemia/reperfusion
CM: Cardiac myocytes
GLP-1: Glucagon-like peptide-1
GPCRs: G-protein-coupled receptor
Ex-4: Exendin-4
LV: Left ventricle
AAR: Area at risk
IS: Infarct size
cTnI: Cardiac troponin I
DPP-4: Dipeptidyl-peptidase-4
HEK: Human embryonic kidney
T2DM: Type 2 diabetes mellitus
